# A novel approach to determine aortic valve area with phase-contrast cardiovascular magnetic resonance

**DOI:** 10.1186/s12968-021-00838-w

**Published:** 2022-01-06

**Authors:** Felix Troger, Ivan Lechner, Martin Reindl, Christina Tiller, Magdalena Holzknecht, Mathias Pamminger, Christian Kremser, Johannes Schwaiger, Sebastian J. Reinstadler, Axel Bauer, Bernhard Metzler, Agnes Mayr, Gert Klug

**Affiliations:** 1 University Clinic of Radiology, Medical University of Innsbruck, Anichstrasse 35, 6020 Innsbruck, Austria; 2 University Clinic of Internal Medicine III, Cardiology and Angiology, Medical University of Innsbruck, Anichstrasse 35, 6020 Innsbruck, Austria; 3 Department of Internal Medicine, Academic Teaching Hospital Hall in Tirol, Hall in Tirol, Austria

**Keywords:** Aortic stenosis, Cardiac magnetic resonance imaging, Phase-contrast-CMR, Valvular heart disease

## Abstract

**Background:**

Transthoracic echocardiography (TTE) is the diagnostic routine standard for assessing aortic stenosis (AS). However, its inaccuracies in determining stroke volume (SV) and aortic valve area (AVA) call for a more precise and dependable method. Phase-contrast cardiovascular magnetic resonance imaging (PC-CMR) is a promising tool to push these boundaries. Thus, the aim of this study was to validate a novel approach based on PC-CMR against the gold-standard of invasive determination of AVA in AS compared to TTE.

**Methods:**

A total of 50 patients with moderate or severe AS underwent TTE, cardiac catheterization and CMR. AVA via PC-CMR was determined by plotting momentary flow across the valve against flow-velocity. SV by CMR was measured directly via PC-CMR and volumetrically using cine-images. Invasive SV and AVA were determined via Fick-principle and Gorlin-formula, respectively. TTE yielded SV and AVA using continuity equation. Gradients were calculated via the modified Bernoulli-equation.

**Results:**

SV by PC-CMR (85 ± 31 ml) correlated strongly (r: 0.73, p < 0.001) with cine-CMR (85 ± 19 ml) without significant bias (lower and upper limits of agreement (LLoA and ULoA): − 41 ml and 44 ml, p = 0.83). In PC-CMR, mean pressure gradient correlated significantly with invasive determination (r: 0.36, p = 0.011). Mean AVA, as determined by PC-CMR during systole (0.78 ± 0.25 cm^2^), correlated moderately (r: 0.54, p < 0.001) with invasive AVA (0.70 ± 0.23 cm^2^), resulting in a small bias of 0.08 cm^2^ (LLoA and ULoA: − 0.36 cm^2^ and 0.55 cm^2^, p = 0.017). Inter-methodically, AVA by TTE (0.81 ± 0.23 cm^2^) compared to invasive determination showed similar correlations (r: 0.58, p < 0.001 with a bias of 0.11 cm^2^, LLoA and ULoA: − 0.30 and 0.52, p < 0.001) to PC-CMR. Intra- and interobserver reproducibility were excellent for AVA (intraclass-correlation-coefficients of 0.939 and 0.827, respectively).

**Conclusions:**

Our novel approach using continuous determination of flow-volumes and velocities with PC-CMR enables simple AVA measurement with no bias to invasive assessment. This approach highlights non-invasive AS grading through CMR, especially when TTE findings are inconclusive.

**Supplementary Information:**

The online version contains supplementary material available at 10.1186/s12968-021-00838-w.

## Background

**Fig. 1:**
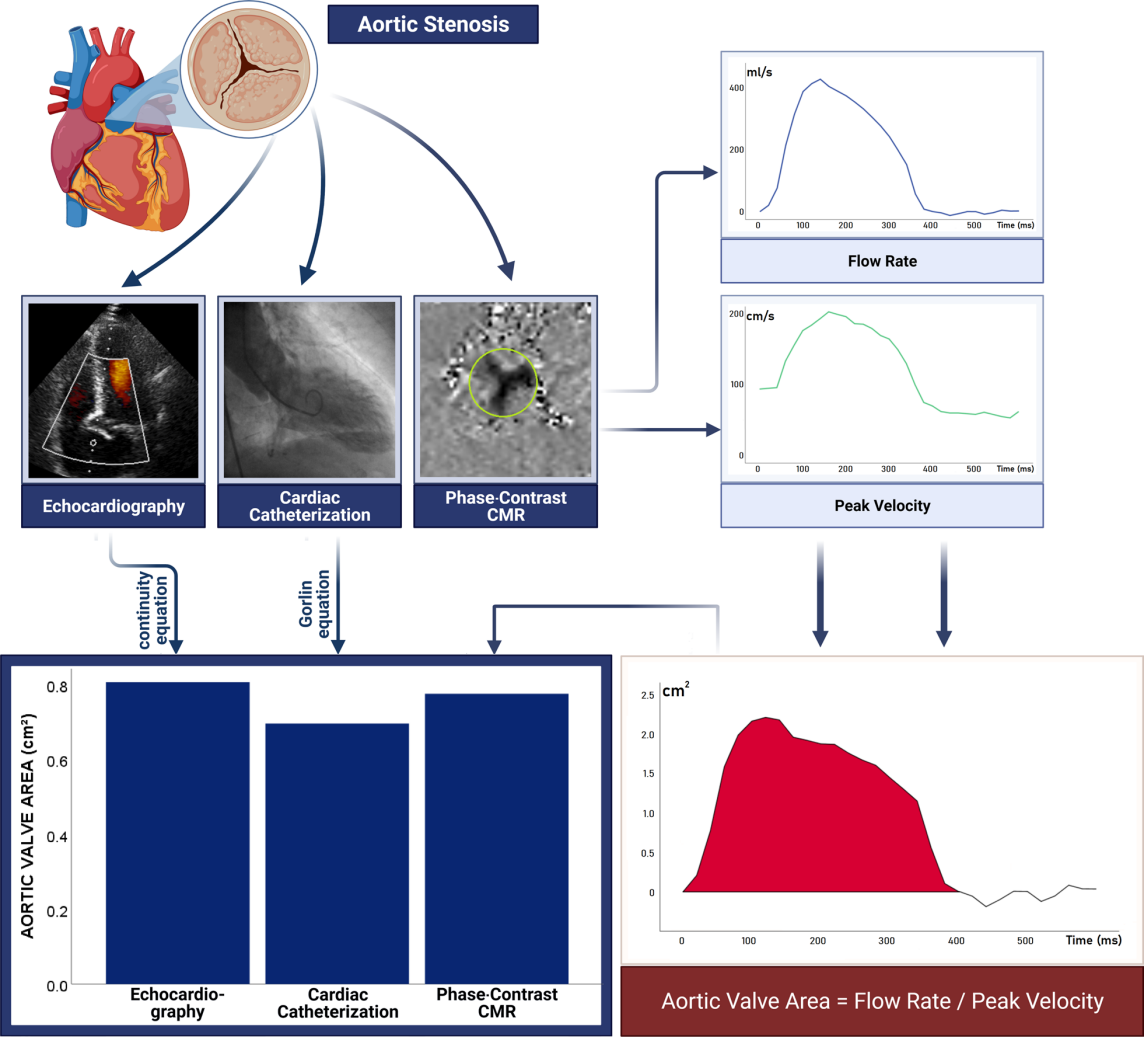
Several approaches to determine aortic valve area, with phase-contrast-cardiovascular magnetic resonance (CMR) yielding solid values comparable to invasive measurement. (Illustration created using biorender.com)

Aortic stenosis (AS) is regarded the most common valvular heart disease in the Western world, with an estimated prevalence of 3% in patients aged ≥ 75 years [1]. Its hemodynamic severity is best characterized by the transaortic maximum velocity, when transaortic volume flow-rates are normal. However, in patients with low transvalvular flow-rates due to either left ventricular (LV) systolic dysfunction or due to a small hypertrophied LV, diagnostic and management challenges differ from AS patients with high gradient and velocity [2]. Although cardiac catheterization with invasive hemodynamic evaluation was historically the gold-standard for diagnosis of severe valvular heart disease [3], nowadays, due to the risks of invasive measurement and advances in noninvasive methods, transthoracic echocardiography (TTE) is considered the first-choice imaging modality to diagnose and classify AS [4]. However, in recent years, cardiovascular magnetic resonance imaging (CMR) has emerged to be a useful tool in characterizing valvular heart disease, and a good and reliable alternative to invasive techniques such as cardiac catheterization and transesophageal echocardiography (TEE) [5]. The equivalence of valve area measurements in TEE and CMR has recently been shown in a meta-analysis by Woldendorp et al., providing aortic valve area (AVA) measurements with virtually no bias [6]. Compared to TTE, most studies showed a slight overestimation of both AVA and stroke volume index (SVI) using CMR, but stated good correlation and concordance [7–9]. Regarding prognostic and therapeutic impact, precise grading of AS severity is absolutely necessary. However, CMR and especially phase-contrast-CMR (PC-CMR) are still used far below their capabilities in AS, and comparison to cardiac catheterization is still lacking. As one main application field of PC-CMR lies in the quantification of flow-volumes and flow-velocities, we deduced that AVA values could be directly determined by calculating the ratio of these two parameters. Thus, determination of AVA would be possible at each moment of the cardiac cycle. Nevertheless, a comparable formula has not yet been assessed in AS, just like PC-CMR studies in AS are still scarce goods. The aims of our study were, therefore, as follows: (1) to compare stroke volumes (SV) as determined by PC-CMR to the ‘gold-standard’ cine-CMR; (2) to determine pressure gradients across the aortic valve (AV) via PC-CMR; and (3) to validate a novel approach of determining AVA by comparing it to the ‘gold-standard’ cardiac catheterization (Fig. 1).

## Methods

### Study population

**Fig. 2:**
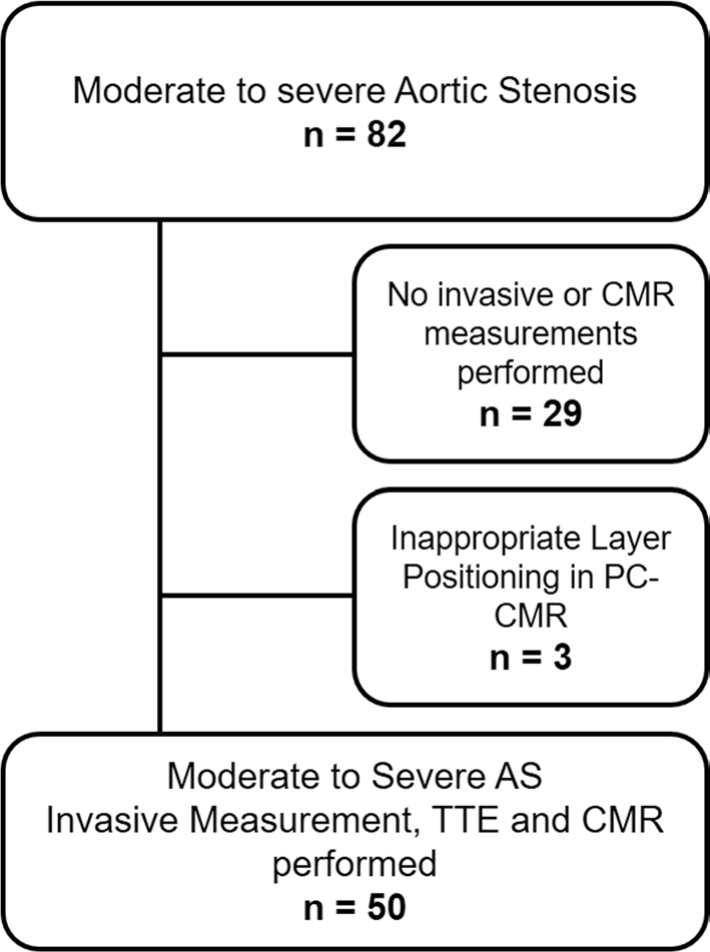
Flowchart of included and excluded participants. *CMR* cardiovascular magnetic resonance imaging, *PC-CMR* phase-contrast CMR, *TTE* transthoracic echocardiography

The study population consisted of 50 consecutive patients with moderate (n = 6) or severe (n = 44) AS that were referred for further investigation; a flowchart of participant in- and exclusion is shown in Fig. 2. Patients were enrolled in a prospective clinical research study at the University Clinic for Cardiology and Angiology Innsbruck, Austria. All patients underwent cardiac catheterization, TTE and CMR. Twenty-four patients had low-flow states as determined by TTE. We excluded patients younger than 18 years of age and those with arrhythmias during CMR scans, contraindications for cardiac catheterization or CMR and patients with prior rheumatic or infectious heart disease. Prior to study inclusion, written informed consent was given by all participants. The study was designed and conducted in compliance with the Declaration of Helsinki and received approval by the local research ethics committee.

### Cardiovascular magnetic resonance

All CMR scans were performed on a 1.5 T clinical CMR scanner (MAGNETOM Avantofit; Siemens Healthineers AG, Erlangen, Germany) 16 days (IQR 1–30) after cardiac catheterization. High-resolution cine-images in long- and short axis covering the LV (10–12 slices) were acquired using a balanced steady state free precession (bSSFP) sequence with retrospective electrocardiographic (ECG) gating (slice thickness: 8 mm, interslice gap: 2 mm, echo time: 1.19 ms, repetition time: 2.83 ms, 22 lines per segments, temporal frame duration: 62.26 ms, frame rate: 25 frames per second, flip angle: 70°, field of view: 380 × 310 mm, matrix: 320 × 260, voxel size: 2.6 × 1.8 × 8.0 mm^3^, parallel imaging mode: GRAPPA (generalized autocalibrating partial parallel acquisition) with acceleration factor 2). To quantify blood flow across the aortic valve via PC-CMR, a modified free-breathing, velocity-encoded phase-contrast-protocol with a spatial resolution of 1.3 × 1.3 × 8 mm was applied without through-plane correction for cardiac motion. Velocity encoding ranged from 300 to 800 cm/s, with 500 cm/s being used most often (n = 25). Retrospective ECG-triggering with 20 (n = 1), 50 (n = 42) or 128 (n = 7) phases per cardiac cycle was applied. Repetition time was 13.56 ms, echo time was 2.62 ms. The mean heart rate (HR) during PC-CMR measurements was 68 ± 13 beats per minute, resulting in a mean reconstructed temporal resolution of 17 ms. One (n = 11), two (n = 2), three (n = 16) or five (n = 21) slices were set perpendicular to the aortic root to measure through-plane flow.

cvi42 software (Circle Cardiovascular Imaging, Calgary, Alberta, Canada) was used for post-processing analyses with semi-automatic detection of LV endo- and epicardial borders. Papillary muscles were excluded from LV myocardial mass (LVM) and included in the LV volume. Myocardial mass, end-diastolic volume (EDV) and end-systolic volume (ESV) were then divided by the body surface area (BSA) [m^2^] to obtain indexed values (LVMI, EDVI and ESVI, respectively). To calculate BSA, the Du Bois formula was used [10]. In 4 patients, the obtained cine-image stacks were not suitable for volumetric measurements.

**Fig. 3:**
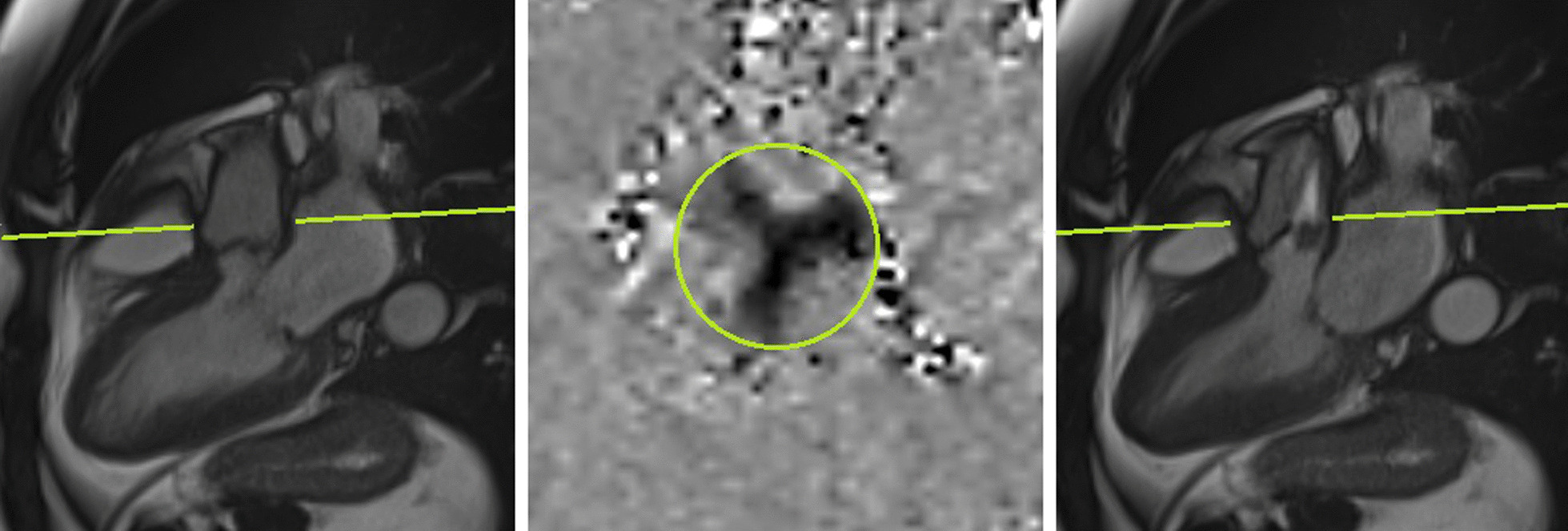
Cine-3-Chamber CMR (left end-diastolic, right end-systolic) and phase-contrast-CMR (end-systolic, middle) in the displayed layer of the aortic valve of a patient with severe aortic stenosis

If available, for our PC-CMR measurements, the uppermost layer that still included the AV during systole was used, which was chosen in synopsis with three-chamber cine-stacks, shown in Fig. 3.

**Fig. 4:**
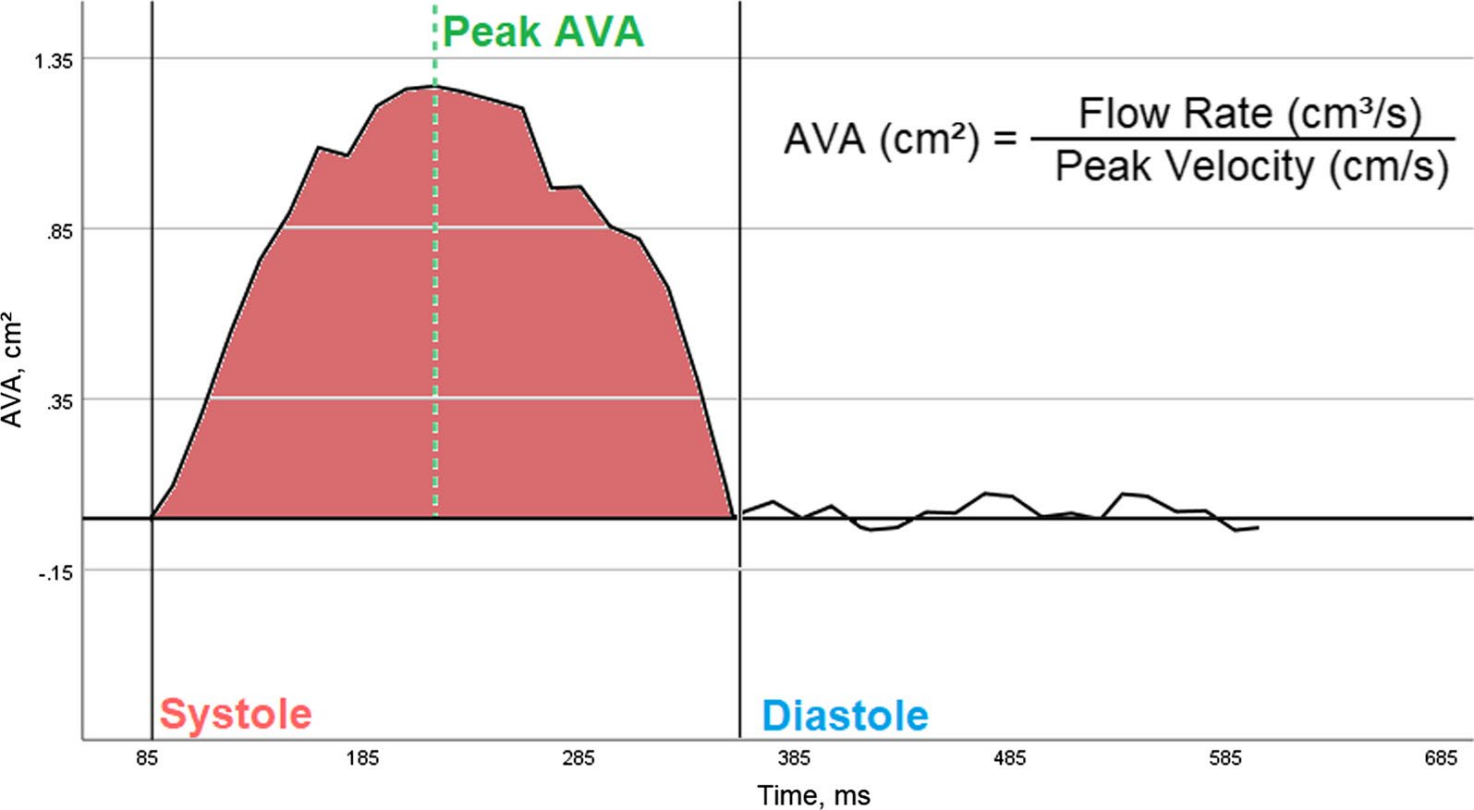
Calculation of aortic valve area (AVA) with simultaneous measurement of flow-volumes and flow-velocities across the aortic valve. According to the formula given above AVA can be calculated continuously over the whole cardiac cycle.The graph shows an example of 50 phases across the cardiac cycle of a patient with a mean AVA of 0.92 cm^2^ and a stroke volume of 44 ml by PC-CMR. *AVA* aortic valve area, *PC-CMR* phase-contrast cardiovascular magnetic resonance imaging

Using cvi42 software (Circle Cardiovascular Imaging), we computed the momentary flow-volume rate and momentary peak velocity at each measuring point (Fig. 4); measuring range for peak velocity was defined as the single “fastest” pixel without using neighbouring pixels. Contours of the AV orifice area were drawn manually on all images of the chosen slice, and flow [ml/s] throughout the cardiac cycle was measured using velocity values of the corresponding velocity-encoded images. LV-SV was defined as forward blood flow through the AV over the cardiac cycle minus reverse blood flow in the same layer. HR was determined from the mean RR interval of cine-stacks and PC-CMR, respectively. SVI was calculated by dividing SV by BSA. In 12 patients (24%), a phase unwrapping algorithm was used to salvage datasets with obvious velocity aliasing.

Pressure gradients were calculated according to the modified Bernoulli-equation [11]; for mean pressure gradient (MPG), mean velocity over the whole systolic phase was used; for peak pressure gradient (PPG), the peak velocity was used.

In PC-CMR, AVA was calculated according to Eq. 1, in order to obtain a momentary AVA for every moment of the cardiac cycle. Finally, AVA during systole was calculated as mean AVA over the whole systolic cycle.

1 $${{AVA}}\left( {{{cm}}^{2} } \right) = {\raise0.7ex\hbox{${{{Flow}}\;{{Rate}}\left( {\frac{{{{ml}}}}{{{s}}}} \right)}$} \!\mathord{\left/ {\vphantom {{{{Flow}}\;{{Rate}}\left( {\frac{{{{ml}}}}{{{s}}}} \right)} {{{Peak}}\;{{Velocity}}\left( {\frac{{{{cm}}}}{{{s}}}} \right)}}}\right.\kern-\nulldelimiterspace} \!\lower0.7ex\hbox{${{{Peak}}\;{{Velocity}}\left( {\frac{{{{cm}}}}{{{s}}}} \right)}$}}$$

To perform planimetry of the AVA, three cine images were acquired orthogonal to the aortic root (slice thickness: 5 mm, no interslice gap). The slice, which exactly recorded the valve opening was used for planimetry of the anatomical AVA.

Additionally, we used the Hakki-formula to compute AVA. This equation represents a simplified version of the Gorlin-formula, calculated as the ratio of cardiac output (CO) and the square root of MPG [12, 13].

### Echocardiography

All patients underwent a comprehensive TTE examination in our tertiary echo lab, including assessment of the AV by measuring SV, pressure gradients and AVA via the continuity equation and LV ejection fraction (LVEF) by the Simpson method. Patients were then divided into two groups according to their flow status—group 1 had low-flow states with a SVI ≤ 35 ml/m^2^, group 2 had normal-flow states with a SVI > 35 ml/m^2^.

### Cardiac catheterization

All patients were referred to left and right heart catheterization. A 7-French Swan-Ganz-catheter was routinely used for hemodynamic measurements. LV end-systolic and end-diastolic pressures were recorded. CO was determined according to Fick principle [14], SV was calculated according to Eq. 2.

2 $$SV \left({\rm ml}\right)=\left. Cardiac Output \left(\frac{l}{\mathrm{min}}\right) \right/ heart rate \left(\frac{1}{\mathrm{min}}\right)*1000$$

Indexing SV by the BSA yielded SVI. AV gradients were assessed by simultaneous measurement of LV and aortic pressures by a double-lumen-catheter placed in the LV and the aortic root. Invasive MPG was calculated via the area under these pressure curves and the peak‐to‐peak gradient pressure by the difference between LV end-systolic and systolic arterial pressure. Systolic and diastolic arterial pressure were measured invasively. AVA was then calculated using the Gorlin-formula (Eq. 3).

3 $$AVA\left( {cm^{2} } \right) = \frac{{{{\left( {Cardiac\;Output \left( {\frac{l}{\min }} \right)*1000} \right)} \mathord{\left/ {\vphantom {{\left( {Cardiac\;Output \left( {\frac{l}{\min }} \right)*1000} \right)} {\left( {systolic ejection period \left( \frac{s}{beat} \right)*heart rate\left( {\frac{1}{\min }} \right)} \right)}}} \right. \kern-\nulldelimiterspace} {\left( {systolic ejection period \left( \frac{s}{beat} \right)*heart rate\left( {\frac{1}{\min }} \right)} \right)}}}}{{44.5*\sqrt {mean pressure gradient \left( {mmHg} \right)} }}$$

In a small subgroup of 17 patients, SV and AVA were additionally determined via thermodilution. None of our patients underwent AV intervention during the study period.

### Statistical analysis

SPSS (version 26.0; Statistical Package for the Social Sciences, International Business Machines, Inc., Armonk, New York, USA) was used for statistical analyses. Kolmogorov–Smirnov-test was applied to test for normal distribution. All results for continuous variables are expressed as means ± standard deviation (SD) if normal distribution was given, or as medians with interquartile range if not. A p-value < 0.05 was considered as statistically significant. To evaluate the agreement between two methods, Pearson correlation as well as Bland–Altman analysis were used; limits of agreement (LoA) were defined as mean difference plus (upper LoA, ULoA) or minus (lower LoA, LLoA) 1.96 × SD. Bias was defined as mean difference between methods. Pearson’s correlation was interpreted as negligible (< 0.2), low (0.2–0.39), moderate (0.4–0.59), strong (0.6–0.79) and very strong (≥ 0.8) [15]. Receiver operating characteristic (ROC) analysis was performed to assess the discriminative power of CMR in discriminating between moderate and severe AS. Area under the curve (AUC) values were interpreted according to Rice and Harris [16]. Dichotomization of AVA via CMR was performed by using the Youden-index and, regarding its impact on therapeutic decision-making, the cut-off with highest specificity was used. Intra- and interobserver variability were evaluated by two observers blinded to each other’s results in all study participants, using a two-way mixed-effects model with intraclass correlation coefficients (ICC).

## Results

### Baseline patient characteristics

**Fig. 5:**
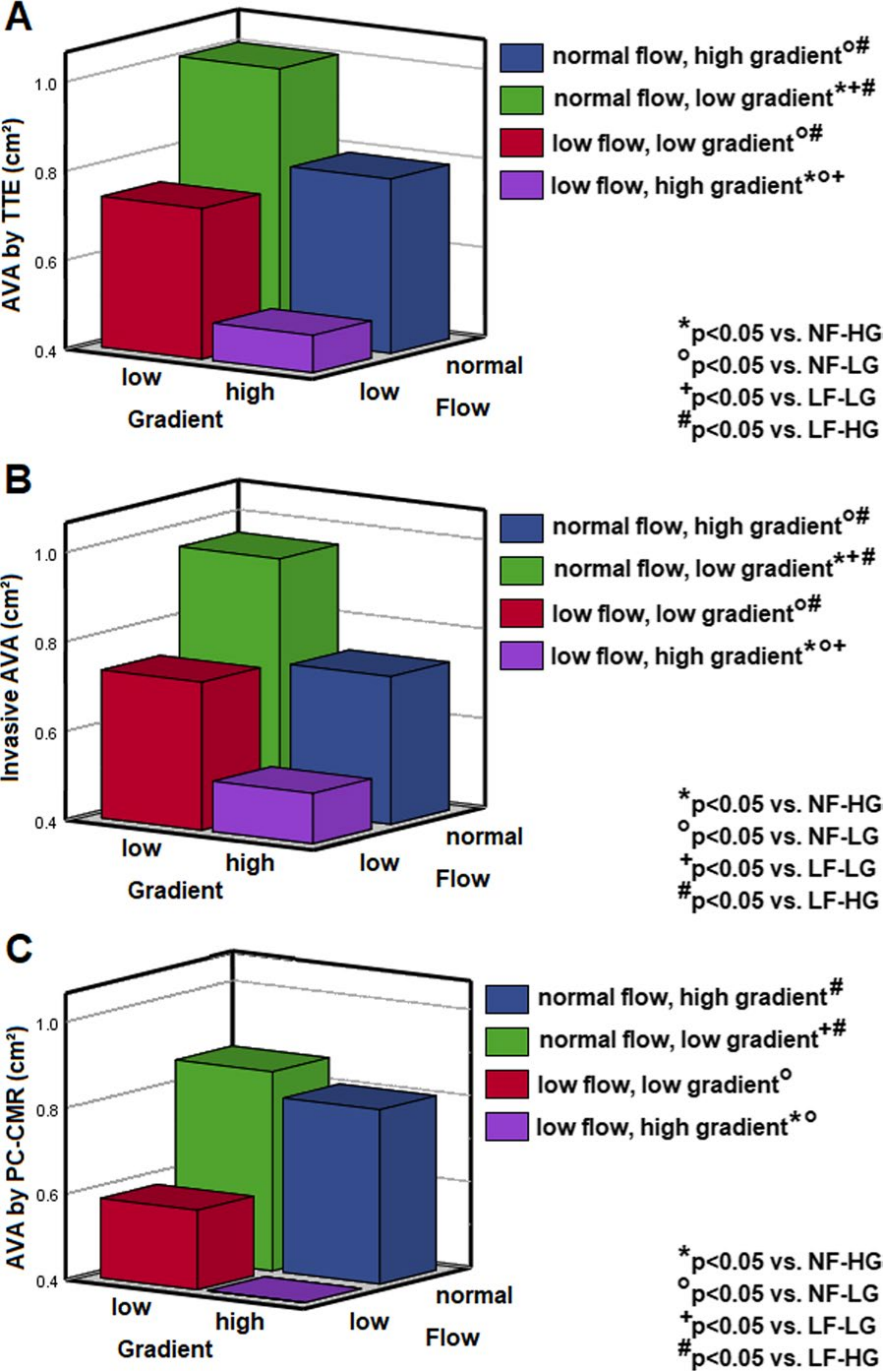
AVA by the respective modality classified according to flow and gradient states. Panel **A** AVA by TTE. Panel **B** Invasive AVA. Panel **C** AVA by PC-CMR. *AVA* aortic valve area, *HG* high gradient, *LF* low-flow, *LG* low gradient, *NF* normal-flow, *PC-CMR* phase-contrast-cardiovascular magnetic resonance, *TTE* transthoracic echocardiography

Mean age of the overall cohort was 71 ± 9 years, 42% (n = 21) were female. All patients underwent CMR 28 days (IQR 15–62) after TTE and 16 days (IQR 1–31) after cardiac catheterization. During these intervals, no patient showed clinical signs of deterioration or complained of an increase in symptom intensity or frequency. In 5 patients (10%), blood pressure medication was initiated or adapted before CMR was performed. Thirty-one patients (62%) had tricuspid AV, 19 (38%) had bicuspid AV. Thirty patients (60%) had aortic regurgitation in TTE, of whom 19 had first-degree and 11 had second-degree regurgitation. The baseline patient characteristics are summarized in Table 1. Patients were then split into two groups according to their flow state in TTE (group 1 with low-flow, n = 24; group 2 with normal-flow, n = 26). Results of echocardiography, cardiac catheterization and CMR according to different flow states are displayed in Table 2. AVA measures by the respective modality are shown in Fig. 5 according to flow and gradient states. Key results of correlation values and intermethodical biases are listed in the following paragraphs, details are listed in Table 3, corresponding Bland–Altman plots are shown in the Additional file 1.

**Table 1:** Baseline patient characteristics

	All patients (n = 50)	Low-flow (n = 24)	Normal-flow (n = 26)	p-value
Age, years	71 ± 9	72 ± 10	70 ± 9	0.662
Female, n (%)	21 (42)	9 (38)	12 (46)	0.536
Body mass index, kg/m^2^	27 ± 5	27 ± 5	26 ± 5	0.477
Moderate AS, n (%)	6 (12)	1 (4)	5 (19)	0.101
Severe AS, n (%)	44 (88)	23 (96)	21 (81)	0.101
Bicuspid valve, n (%)	19 (38)	8 (33)	11 (42)	0.514
Smokers, n (%)	15 (30)	10 (42)	5 (19)	0.119
Pack years	43 ± 184	70 ± 254	12 ± 20	0.215
Hypertension, n (%)	37 (74)	20 (83)	17 (65)	0.212
Dyslipidemia, n (%)	36 (72)	17 (71)	19 (73)	0.682
Diabetes mellitus, n (%)	11 (22)	8 (33)	3 (12)	0.074
AS symptoms	44 (88)	21 (88)	23 (89)	0.603
Vertigo	17 (34)	9 (38)	8 (31)	0.686
Syncope	7 (14)	2 (8)	5 (19)	0.220
NYHA class, n (%)				
I	2 (4)	1 (4.2)	1 (3.8)	0.302
II	12 (24)	3 (12.5)	9 (34.6)	0.056
III	29 (58)	17 (70.8)	12 (46.2)	0.104
IV	7 (14)	3 (12.5)	4 (15.4)	0.726
CCS class, n (%)				
I	6 (12)	2 (8.3)	4 (15.4)	0.672
II	21 (42)	8 (33.3)	13 (50)	0.187
III	18 (36)	10 (41.7)	8 (30.8)	0.483
IV	5 (10)	4 (16.7)	1 (3.8)	0.143
Coronary artery disease, n (%)	34 (68)	19 (79)	15 (58)	0.204
Atrial fibrillation, n (%)	7 (14)	6 (25)	1 (4)	**0.036**
Lab parameters				
eGFR, ml/min/1.73 m^2^	58 ± 5	57 ± 6	58 ± 4	0.792
NT-proBNP, ng/l	1184 ± 2836	1438 ± 3786	949 ± 1566	0.472
Troponin T, ng/l	15 ± 9	17 ± 10	14 ± 7	0.472
Total cholesterole, mg/dl	176 ± 43	168 ± 44	183 ± 41	0.194

p-values in bold indicate statistical significance (p < 0.05)

*AS* aortic stenosis, *CCS* Canadian Cardiovascular Society angina grading scale, e*GFR* estimated glomerular filtration rate, *NT-proBNP* N-terminal prohormone of brain natriuretic peptide, *NYHA* New York Heart Association functional classification

**Table 2:** TTE, invasive and CMR measurements

TTE, n	All patients (n = 50)	Low-flow (n = 24)	Normal-flow (n = 26)	p-value
Ejection fraction, %	60 ± 9	58 ± 10	62 ± 8	0.137
SVI (continuity equation), ml/m^2^	37 ± 11	29 ± 5	45 ± 7	** < 0.001**
MPG, mmHg	37 ± 17	30 ± 11	42 ± 19	**0.010**
PPG, mmHg	58 ± 25	49 ± 17	67 ± 27	**0.005**
AVA (continuity equation), cm^2^	0.84 ± 0.23	0.71 ± 0.19	0.90 ± 0.22	**0.002**
Aortic regurgitation, n (%)	30 (60)	14 (58)	16 (62)	0.624
Mitral regurgitation, n (%)	40 (80)	20 (83)	20 (77)	0.258
*Cardiac catheterization, n*	**50**	**24**	**26**	
SVI (Fick), ml/m^2^	30 ± 14	28 ± 5	35 ± 6	**0.005**
MPG, mmHg	41 ± 19	37 ± 12	45 ± 23	0.139
PPG, mmHg	48 ± 27	41 ± 14	54 ± 34	0.081
AVA (Fick), cm^2^	0.70 ± 0.23	0.68 ± 0.17	0.71 ± 0.27	0.583
*Cine-CMR, n*	**46**	**23**	**23**	
LV ejection fraction, %	65 ± 15	61 ± 18	68 ± 9	0.297
SVI, ml/m^2^	45 ± 9	41 ± 9	50 ± 7	**0.001**
EDVI, ml/m^2^	72 ± 23	70 ± 30	75 ± 15	0.468
ESVI, ml/m^2^	28 ± 22	31 ± 30	25 ± 12	0.324
LVMI, g/m^2^	81 ± 22	79 ± 20	83 ± 24	0.668
PC CMR, n	**50**	**24**	**26**	
SVI, ml/m^2^	45 ± 14	41 ± 13	49 ± 15	**0.046**
MPG, mmHg	22 ± 10	19 ± 9	24 ± 10	**0.042**
PPG, mmHg	64 ± 29	56 ± 28	70 ± 29	0.064
AVA, cm^2^	0.78 ± 0.25	0.79 ± 0.23	0.76 ± 0.27	0.736

*AVA* aortic valve area, *CMR* cardiovascular magnetic resonance, *cont. equ.* continuity equation, *EDVI* end-diastolic volume index, *ESVI* end-systolic volume index, *LVMI* left ventricular mass index, *MPG* mean pressure gradient, *PC-CMR* phase-contrast-cardiovascular magnetic resonance, *PPG* peak pressure gradient, *SVI* stroke volume index, *TTE* transthoracic echocardiography

**Table 3:** Correlation values and intermethodical biases

	Pearson’s r	p-value	Bias	LLoA	ULoA	p-value
Stroke volume						
*vs. cine-CMR*						
PC-CMR	0.730	< 0.001	0.7 ml	− 41 ml	44 ml	0.829
TTE	0.504	< 0.001	16 ml	− 24 ml	55 ml	< 0.001
*vs. Invasive measurement (Fick)*						
PC-CMR	0.697	< 0.001	30.4 ml	− 17 ml	76 ml	< 0.001
TTE	0.478	0.001	13 ml	− 22 ml	49 ml	< 0.001
*vs. Invasive measurement (thermodilution)*						
PC-CMR	0.525	0.037	9 ml	− 47 ml	70 ml	0.159
TTE	0.484	0.057	6 ml	− 43 ml	56 ml	0.322
Mean pressure gradient						
*vs. Invasive measurement*						
PC-CMR	0.358	0.011	19 mmHg	− 16 mmHg	54 mmHg	< 0.001
TTE	0.755	< 0.001	3 mmHg	− 21 mmHg	28 mmHg	< 0.001
Peak pressure gradient						
*vs. Invasive measurement*						
PC-CMR	0.328	0.020	17 mmHg	− 46 mmHg	80 mmHg	< 0.001
TTE	0.719	< 0.001	10 mmHg	− 26 mmHg	49 mmHg	< 0.001
*vs. TTE*						
PC-CMR	0.376	0.007	− 5 mmHg	− 54 mmHg	65 mmHg	0.249
Aortic valve area						
*vs. Invasive measurement (Fick)*						
PC-CMR	0.544	< 0.001	0.08 cm^2^	− 0.36 cm^2^	0.54 cm^2^	0.017
TTE	0.580	< 0.001	0.11 cm^2^	− 0.30 cm^2^	0.52 cm^2^	< 0.001
*vs. TTE*						
PC-CMR	0.366	0.009	0.03 cm^2^	− 0.50 cm^2^	0.56 cm^2^	0.414
*vs. Invasive measurement (thermodilution)*						
PC-CMR	0.773	0.001	0.05 cm^2^	− 0.35 cm^2^	0.44 cm^2^	0.409
TTE	0.557	0.039	0.05 cm^2^	− 0.46 cm^2^	0.56 cm^2^	0.459
Invasive (Fick)	0.720	0.004	0.15 cm^2^	− 0.28 cm^2^	0.57 cm^2^	0.024

*CMR* cardiovascular magnetic resonance imaging, *LLoA* lower limit of agreement, *PC-CMR* phase-contrast-CMR, *TTE* transthoracic echocardiography, *ULoA* upper limit of agreement

### Stroke volume

**Fig. 6:**
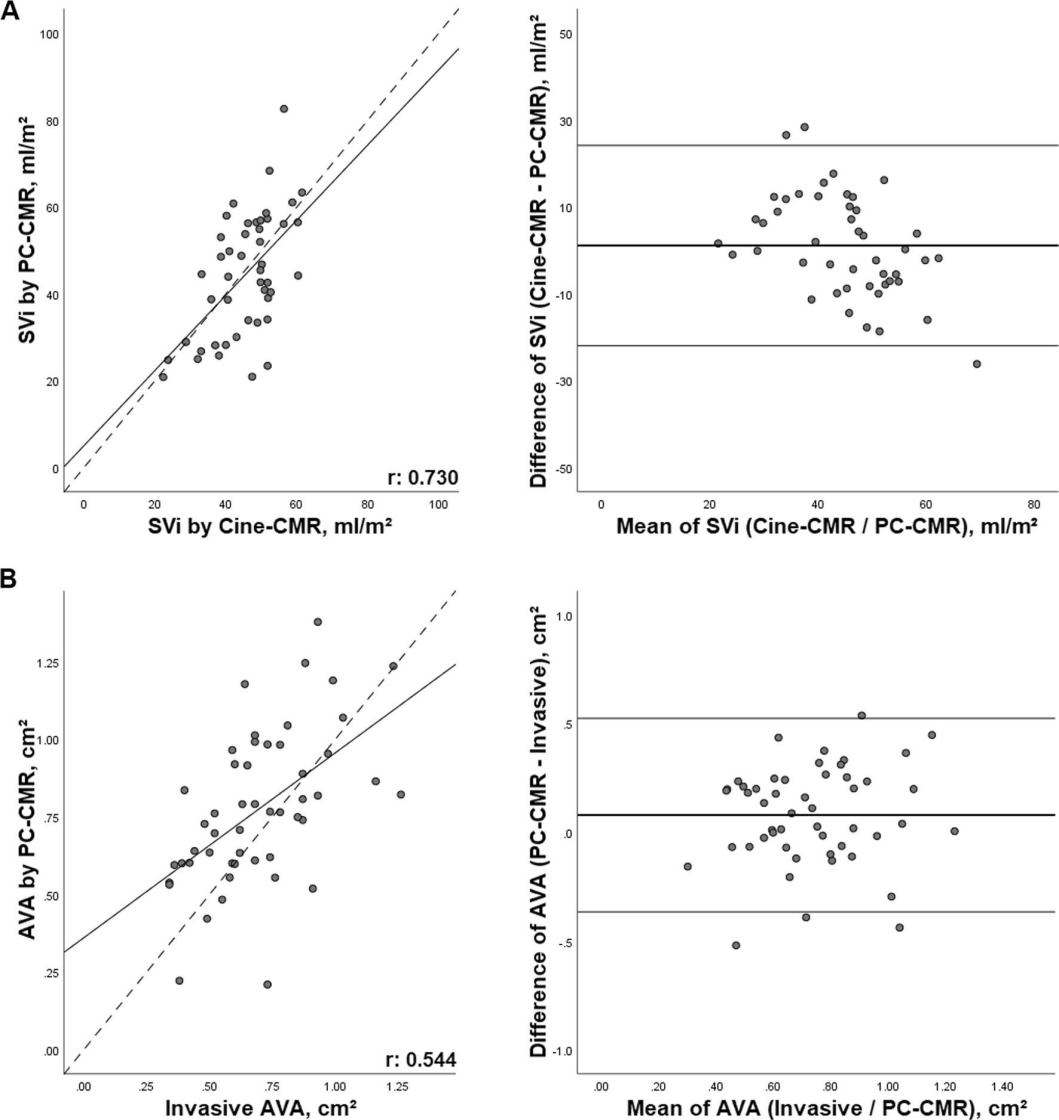
Panel **A** Scatter plot and Bland–Altman-diagram of SVI via cine-CMR and PC-CMR. Panel **B** Scatter plot and Bland–Altman-diagram of AVA invasively and via PC-CMR. *AVA* aortic valve area, *CMR* cardiovascular magnetic resonance imaging, *PC-CMR* phase-contrast-cardiovascular magnetic resonance imaging, *SVI* stroke volume index

SV by PC-CMR (85 ± 31 ml) correlated strongly with volumetric measurements in cine-images (85 ± 19 ml; r: 0.73, p < 0.001) and with invasive determination according to Fick principle (57 ± 13 ml; r: 0.70, p < 0.001). Scatter plot and Bland–Altman-analysis are shown in Fig. 6, panel A.

In TTE, SV had moderate correlation to cine-volumetry (r: 0.50, p < 0.001) and to invasive data (r: 0.48, p = 0.001) according to continuity equation (71 ± 21 ml).

SV determined via thermodilution also strongly correlated with SV measured according to Fick principle, with the former providing higher values. With regard to thermodilution, SV correlated moderately with PC-CMR and moderately but non-significantly with TTE (see Table 3).

ROC-analysis of PC-CMR-derived SVI to distinguish low and normal flow was not significant (AUC 0.651; p = 0.056).

### Pressure gradients

Mean and peak gradients by PC-CMR (22 ± 10 mmHg and 64 ± 29 mmHg, respectively) correlated low with invasive measurements (41 ± 19 mmHg and 48 ± 27 mmHg, respectively). PPG by PC-CMR had low correlation with TTE (58 ± 25 mmHg). MPG by TTE (37 ± 17 mmHg) correlated strongly with cardiac catheterization, as did PPG by TTE (58 ± 25 mmHg). Correlation values and biases are listed in detail in Table 3.

### Aortic valve area

In PC-CMR, mean AVA during systole (0.78 ± 0.25 cm^2^) showed good correlation (r: 0.54, p < 0.001) with a bias of 0.08cm^2^ (p = 0.017) compared to invasively determined AVA according to the Fick principle (0.70 ± 0.23 cm^2^). Scatter plot and Bland–Altman-analysis are presented in Fig. 6, panel B.

In TTE, mean AVA as measured by the continuity equation (0.84 ± 0.23 cm^2^) correlated moderately (r: 0.58, p < 0.001, bias: 0.11 cm^2^, p < 0.001) to invasive measurement. AVA by TTE correlated low with AVA measured by PC-CMR (0.366, p = 0.009, bias: 0.03 cm^2^, p = 0.41).

Invasively determined AVA values (i.e. Fick principle and thermodilution) correlated strongly with each other, with thermodilution providing higher values. AVA determined via thermodilution showed a good agreement with AVA measured by PC-CMR over the whole systolic cycle and by TTE. A detailed list of correlation values and biases is shown in Table 3.

Defining the invasively measured AVA by the Fick principle as threshold value between moderate and severe AS, ROC-analysis revealed an AUC-value of 0.81 for AVA determined by PC-CMR. Dichotomization of AVA by PC-CMR at a cut-off value of 0.82 cm^2^ (Youden-index) yielded a specificity of 100% (i.e. all moderate AS were classified correctly) and a sensitivity of 67% (i.e. two-thirds of severe AS were classified correctly). At a cut-off value of 1 cm^2^, specificity was 50% and sensitivity 87%.

In TTE, ROC-Analysis showed an AUC of 0.815, with a cut-off at 1.05 cm^2^ (Youden-index) yielding a specificity and sensitivity of 89% and 75%, respectively.

Cine-planimetry yielded AVA values that weakly correlated with invasive determination (planimetric AVA: 0.97 ± 0.37 cm^2^; r: 0.35, p = 0.013, bias: − 0.28 cm^2^, p < 0.001) and AVA by PC-CMR (r: 0.32, p = 0.022, bias: 0.20 cm^2^, p < 0.001).

AVA computed via the Hakki formula (0.76 ± 0.30 cm^2^) showed moderate correlation (r: 0.42, p = 0.002) and a bias of 0.06 cm^2^ (p = 0.15) compared to invasive determination, and a very strong correlation (r: 0.86, p < 0.001) with a bias of 0.02 cm^2^ (p = 0.35) compared to our PC-CMR method.

### Intra and interobserver variabilty

Intra- and interobserver reproducibility of PC-CMR derived measures were excellent for SV (ICC of 0.955 [95%CI 0.921–0.974] and 0.970 [95%CI 0.947–0.983], respectively) as well as for PPG (ICC of 0.918 [95%CI 0.855–0.953] and 0.892 [95%CI 0.811–0.939], respectively) and for AVA (ICC of 0.939 [95%CI 0.892–0.965] and 0.827 [95%CI 0.696–0.902], respectively). Scatter and Bland–Altman plots are shown in the Additional file 1: Figs. S1 (Intraobserver) and S2 (Interobserver).

## Discussion

To the best of our knowledge, the utilization of our novel approach simultaneously represents the first-ever study to compare PC-CMR with invasively derived measurements in AS. The main study finding can be summarized as follows: PC-CMR is able to compute AVA values via an innovative technique by plotting momentary flow across the valve against flow-velocity that are very similar to invasive determination with a negligible bias. Therefore, these results clearly argue for the capability of our novel method using PC-CMR to assess AS in a solid and reliable manner compared to the complex gold-standard of invasive determination.

### Stroke volume

Computation of SV in PC-CMR compared to the gold-standard cine-CMR yielded a strong correlation with no bias. This is in line with previous data of our and other study groups, reporting a strong agreement between these two modalities in healthy subjects and in patients with myocardial infarction, respectively [17, 18]. SV by PC-CMR also showed good correlation with invasive determination, however revealing a clear bias to the invasively measured SV according to the Fick-principle. In contrast, a recent study by Po et al. described a good correlation between PC-CMR and invasive determination of SV applying right heart catheterization in pulmonary hypertension, displaying a bias of 4 ml [19]. Similarly, Mauritz et al. investigated the correlation between SV by PC-CMR in the aorta and via right heart catheterization in pulmonary hypertension, which showed a good agreement with a bias of only 1 ml, albeit in a relatively small cohort [20]. In AS patients, however, no comparable study has been conducted so far. Anyway, due to the altered flow patterns in AS, a structured comparison between a flow-dependent (PC-CMR) and flow-independent (cine) method seems desirable. In our study, cine-CMR served as gold-standard for quantifying SV, so the underestimation of invasive measurements could be due to the complex hemodynamic findings in this disease entity.

TTE assessed SV showed a lower bias, but also less correlation when compared to invasive SV data. Corresponding to several previous studies [9, 18, 21], TTE-assessed SV mostly showed a good agreement to PC-CMR derived SV. Barone-Rochette et al. showed good correlation between SVi by PC-CMR and by TTE; additionally, in that study, PC-CMR was able to confirm low-flow states diagnosed via TTE [9].

In our subgroup analysis with invasively assessed SV via thermodilution, PC-CMR showed good correlation and a distinctly smaller bias, whilst TTE presented a bias of 20 ml and no significant correlation. The discrepancy in SV according to Fick principle compared to thermodilution is in line with selected studies made in patients with pulmonary hypertension, proclaiming no interchangeability between these two measurements, with one of them explicitly recommending thermodilution [22, 23]. Kresoja et al. showed, that especially in elderly patients, SV by the Fick method provided large individual errors, whilst thermodilution served as gold-standard in that study [24]. Anyway, the patients undergoing thermodilution in our study provided good results that sorted well with PC-CMR and cine-CMR measurements; nonetheless, the cohort undergoing thermodilution in our study is quite small, so no general statements can be made.

ROC-analysis of PC-CMR-derived SVI was not significant. However, as was shown by Chin et al., TTE is quite prone to underestimate SV [25], which is why a CMR-based classification seems even more desirable. What is more, a graduated or continual classification of flow-states based on CMR would represent a promising approach and a sensible alternative to the quite arbitrary defined 35 ml/m^2^-threshold. Though, further studies to assess this classification are needed.

### Pressure gradients

In our study, MPG and PPG gradients by PC-CMR showed low correlation to their invasive counterparts, with mean biases of 19 (MPG) and 16 (PPG) mmHg, respectively. In line with our study, in 2003 Caruthers et al. used the modified Bernoulli equation to compute pressure gradients in AS by PC-CMR, yielding a strong correlation compared to TTE, but without quantifying any absolute biases [26]. This discrepancy in correlation values could be due to the waiver of using neighbouring pixels in our study, detecting only the single fastest pixel to compute momentary peak velocity, thus leading to an overestimation of PPG. As opposed to this, MPG was significantly underestimated in our study; a possible explanation could be the general underestimation of peak jet velocity by CMR due to its lower temporal resolution in contrast to ultrasound [5]. A distinct underestimation of MPG between TTE and PC-CMR was also described by Defrance et al., which was even 25 mmHg in a mixed patient cohort and 16 mmHg in AS patients, although higher correlation levels were reported [27]. Sirin et al. described an underestimation of PPG of 12 mmHg in PC-CMR compared to TTE in a small cohort of pediatric AS patients [28]. These divergences could be due to the narrow range of invasive MPG (95% Confidence Interval (CI) 36–46 mmHg) and PPG (95%CI 40–55 mmHg) in our study as opposed to the above-mentioned studies. Moreover, the distinct underestimation of MPG in PC-CMR compared to TTE has been described previously by Garcia et al., grounding this finding (by analogy to ultrasound) on energy loss in AS and on pressure recovery [29–31].

### Aortic valve area

Our main finding was that AVA determined by PC-CMR showed a good compatibility with invasively measured AVA, displaying only a small bias. However, the correlation values yielded in our study still have some room for improvement. The lack of a definite gold-standard in the assessment of AS could be a major reason, implying the possible presence of factors hampering the accuracy of cardiac catheterization (i.e. pressure recovery effects) or TTE (i.e. misalignment of Doppler beam with flow direction) without having an effect on CMR [8, 27]. PC-CMR provided an abundance of methods to determine AVA, but measuring the mean AVA over the whole systolic cycle appears to be the most objectifiable and easiest one, simultaneously yielding the best results. We used cardiac catheterization and calculation of AVA by using the Gorlin-formula as gold-standard to determine AVA. However, the use of this equation to estimate AVA is associated with several pitfalls, as it is directly related to cardiac output, blood viscosity, and turbulent flow. What is more, invasive measurement of the AVA is associated with a substantial risk of cerebral embolism [32]. In 2016, Wong et al. provided a review of 12 papers regarding the comparison between TTE and CMR assessment of AVA in AS, concluding that CMR does not succumb TTE, whilst showing better inter- and intraobserver reliability and greater specificity and sensitivity [33]. Nevertheless, the studies being investigated in that review calculated AVA in CMR very heterogeneously by using either continuity equation [8, 26, 27, 34], cine-planimetry [7, 9, 35, 36], the Hakki formula [27] or planimetry in magnitude images [34]. A fifth approach previously published is planimetry in PC-CMR images, suggesting a better correlation of that method to TEE than cine-planimetry [37]. In our present study, AVA was additionally computed via the Hakki formula and via cine-planimetry, but both methods showed lower correlation and at best a similar bias compared to invasive measurement than our primarily used approach, with both being rather prone to errors.

In comparison to TTE, AVA values obtained by PC-CMR showed only a weak correlation, with PC-CMR demonstrating a lower bias in comparison to the gold-standard of cardiac catheterization. While many studies comparing TTE to CMR in AS reported an overestimation of AVA in CMR [6], our results are in line with a study comparing the continuity equation in TTE to PC-CMR [27], possibly indicating that TTE measurements in AS generally tend to slightly overestimate AVA in AS compared to PC-CMR. One of the major reasons for this finding could be the more exact detection of flow-velocity in PC-CMR, as it is less prone to miss the true peak velocity.

Competing with our PC-CMR approach, Reant et al. described a good concordance between planimetric measurement of AVA in cine-images, direct planimetry in TEE and invasive determination via cardiac catheterization, but a significant bias for TTE [36]. Another study provided evidence for a good concordance between TTE and CMR in a cohort of 31 patients, using continuity equation in both modalities to determine AVA. However, this group described a compensation of an underestimated LV outflow tract area for an overestimated velocity time integral in TTE measurements [8], which appears to be an unstable base for solid calculations. In comparison, our approach of determining AVA via plotting momentary flow-volume across the valve against flow-velocity is an easy-to-apply method that provided stable results with a bearable bias compared to invasive measurement.

In addition to the excellent intra- and interobserver reproducibility shown in our study, the superior interstudy reproducibility of CMR compared to TTE was demonstrated in a study by Grothues et al. [38].

In our subgroup undergoing thermodilution, AVA measured by this method yielded a better correlation and a smaller bias compared to AVA by PC-CMR as opposed to Fick principle. There are hardly any data available comparing these two invasive methods of determining AVA, but by analogy to the determination of SV, higher values of SV in thermodilution lead to larger AVA measures. As mentioned before, this subgroup is too small to justify general statements and studies investigating it on a larger cohort are lacking. However, by tendency, AVA measured via thermodilution appears to be a more stable parameter than AVA calculated via the Fick principle.

## Limitations

Firstly, all of the patients in our study had moderate to severe AS, which makes translation of our results on patients without AS or with only mild AS uncertain. However, it can be assumed that a narrow AV does not have a major impact on the accuracy of PC-CMR, as has already been suggested by Defrance et al. [27]. Another important limitation of this study is the lack of a uniform CMR protocol, leading to PC-CMR measurements in different planes above the AV. Possibly, layer positioning has an unneglectable impact on flow and velocity measurements. Our study group is currently working on further standardization of this. Furthermore, in cardiac catheterization, thermodilution was only performed in a small subset (n = 17) of patients. Another issue is the partially quite long interval between the different examinations. However, according to a study by Nistri et al., mean progression of jet velocity in AS is 0.26 m/s/year [39], which is why this point may not be a major concern in our study. Lastly, in 5 patients, blood pressure medication was changed before CMR scan, which might influence flow dynamics over the AV and furthermore could have an impact on the comparability and objectivity between diagnostic modalities.

## Conclusion

This study introduces a simple and easy-to-use approach to determine AVA in patients with moderate to severe AS via PC-CMR. AVA measurement by continuous determination of flow-volumes and velocities as well as SV determination yielded by PC-CMR were in good agreement with invasive measurement. Thus,

PC-CMR represents a useful tool in diagnosing and characterizing AS that provides a non-invasive alternative to TTE, especially when findings are inconclusive.

## Supplementary Information

**Additional file 1.** **Figure S1.** Intraobserver Variability. Scatter and Bland-Altman plots of stroke volume (Panel A) and aortic valve area (Panel B). AVA: aortic valve area, SV: stroke volume. **Figure S2.** Interobserver Variability. Scatter and Bland-Altman plots of stroke volume (Panel A) and aortic valve area (Panel B). AVA: aortic valve area, Obs: observer, SV: stroke volume. **Figure S3.** Bland-Altman plots of SV and AVA between different modalities. AVA: aortic valve area, PC-CMR: phase-contrast-cardiovascular magnetic resonance imaging, SV: stroke volume, TTE: transthoracic echocardiography.

## Data Availability

The datasets used and/or analysed during the current study are available from the corresponding author on reasonable request.

## Abbreviations

AS: Aortic stenosis
AUC: Area under the curve
AV: Aortic valve
AVA: Aortic valve area
BSA: Body surface Area
bSSFP: Balanced steady state free precession
CI: Confidence interval
CMR: Cardiovascular magnetic resonance imaging
CO: Cardiac output
ECG: Electrocardiogram
EDV: End-diastolic volume
EDVI: End-diastolic volume index
ESV: End-systolic volume
ESVI: End-systolic volume index
HR: Heart rate
ICC: Intraclass correlation coefficient
IQR: Interquartile range
LLoA: Lower limit of agreement
LoA: Limit of agreement
LV: Left ventricle/left ventricular
LVEF: Left ventricular ejection fraction
LVM: Left ventricular mass
LVMI: Left ventricular mass index
MPG: Mean pressure gradient
PC-CMR: Phase-contrast cardiovascular magnetic resonance
PPG: Peak pressure gradient
ROC: Receiver operating characteristics
SD: Standard deviation
SV: Stroke volume
SVI: Stroke volume index
TEE: Transesophageal echocardiography
TTE: Transthoracic echocardiography
ULoA: Upper limit of agreement
