# Supplementary material for: A novel approach to determine aortic valve area with phase-contrast cardiovascular magnetic resonance
Source: J Cardiovasc Magn Reson. 2022 Jan 6;24:7. doi: 10.1186/s12968-021-00838-w (PMC8734220; doi:10.1186/s12968-021-00838-w)
Supplement: Supplementary file 1 — Additional file 1. Figure S1. Intraobserver Variability. Scatter and Bland-Altman plots of stroke volume (Panel A) and aortic valve area (Panel B). AVA: aortic valve area, SV: stroke volume. Figure S2. Interobserver Variability. Scatter and Bland-Altman plots of stroke volume (Panel A) and aortic valve area (Panel B). AVA: aortic valve area, Obs: observer, SV: stroke volume. Figure S3. Bland-Altman plots of SV and AVA between different modalities. AVA: aortic valve area, PC-CMR: phase-contrast-cardiovascular magnetic resonance imaging, SV: stroke volume, TTE: transthoracic echocardiography. [file 12968_2021_838_MOESM1_ESM.docx]

Additional

*
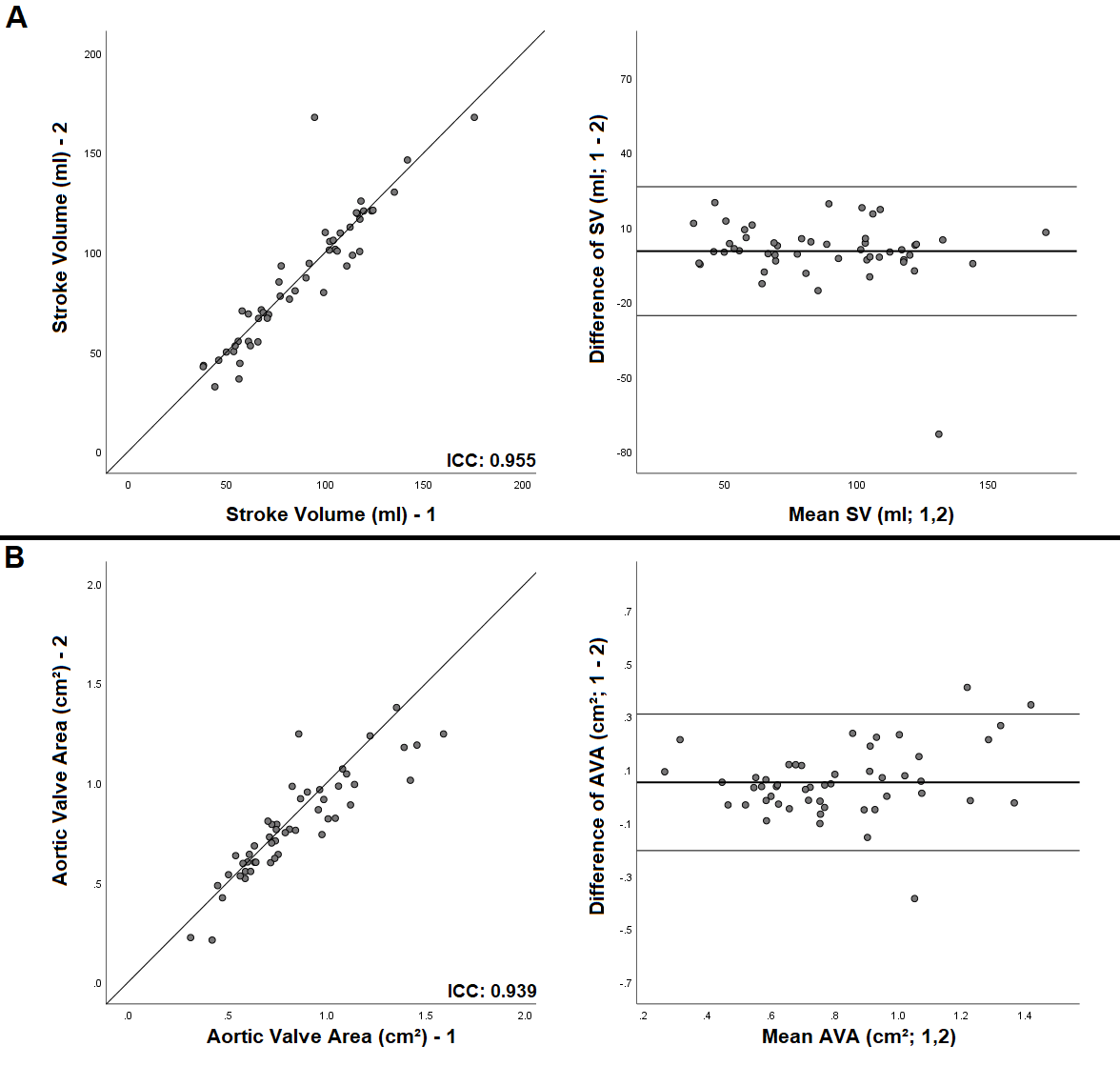
***Additional Figure 1.** Intraobserver Variability. Scatter and Bland-Altman plots of stroke volume (Panel A) and aortic valve area (Panel B). *AVA:* *aortic valve area, SV: stroke volume.*

*
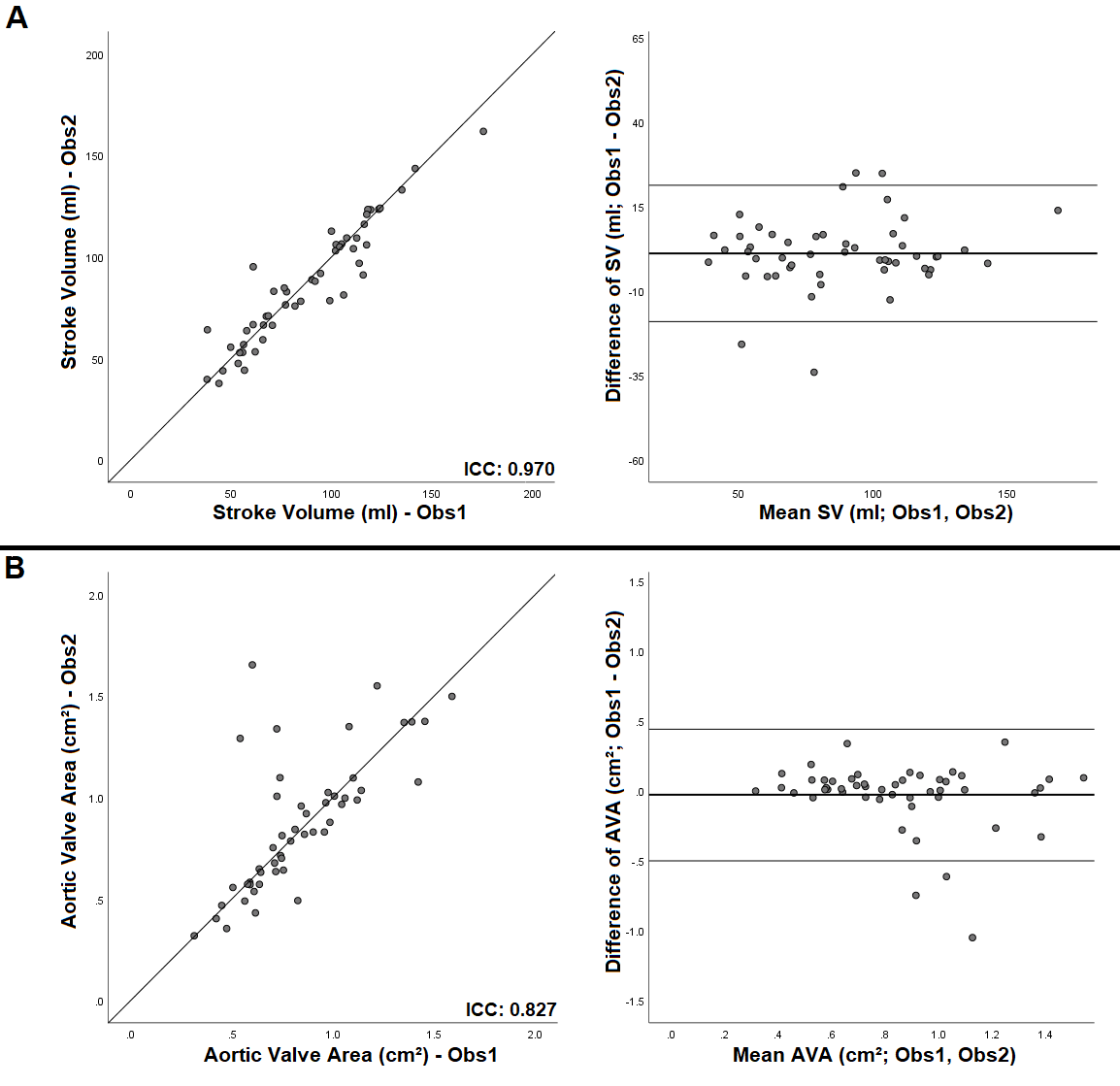
***Additional Figure 2.** Interobserver Variability. Scatter and Bland-Altman plots of stroke volume (Panel A) and aortic valve area (Panel B). *AVA: aortic valve area, Obs: observer, SV: stroke volume.*


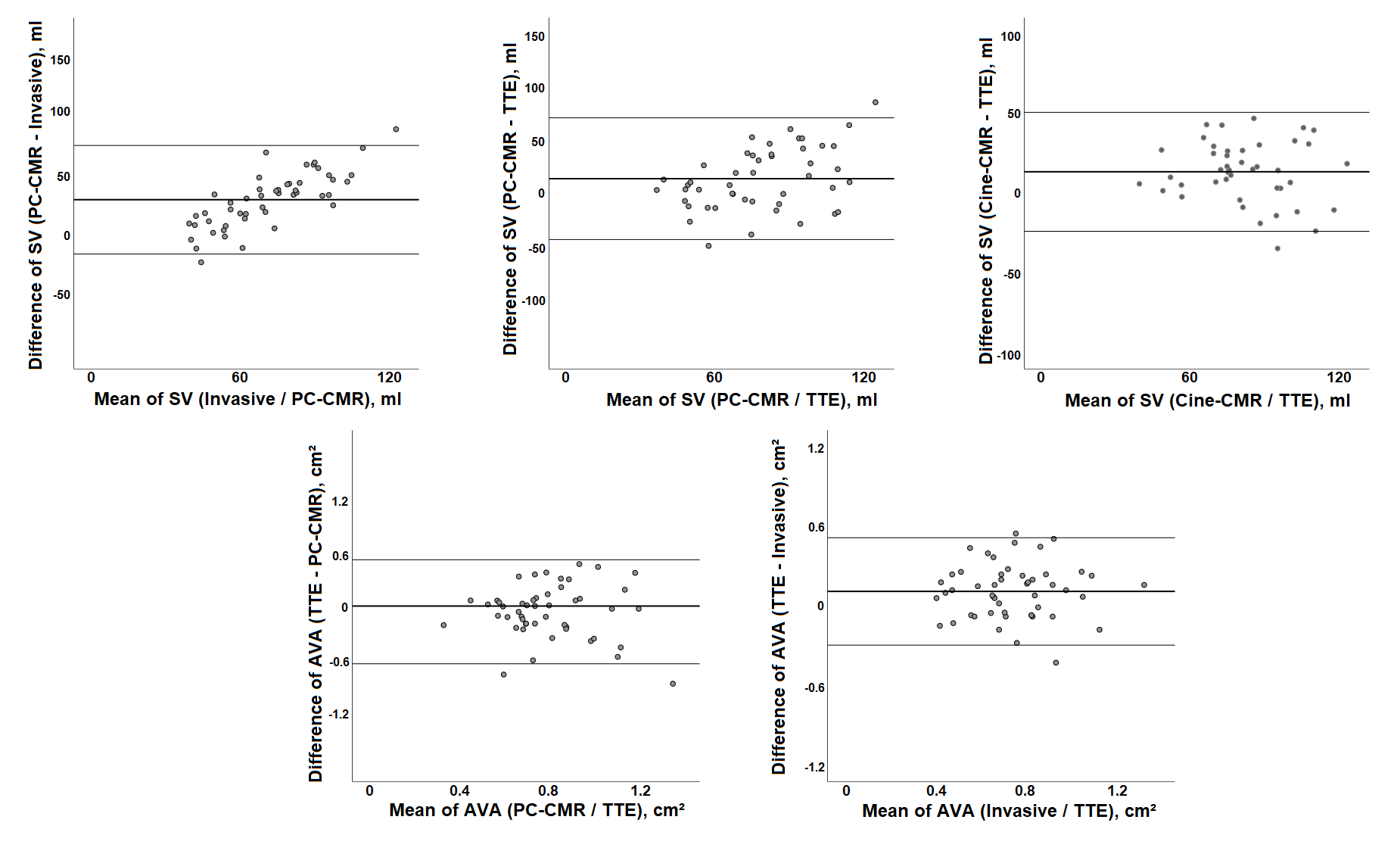
**Additional Figure 3.** Bland-Altman plots of SV and AVA between different modalities. *AVA: aortic valve area, PC-CMR: phase-contrast-cardiovascular magnetic resonance imaging, SV: stroke volume, TTE: transthoracic echocardiography.*
